# Prognostic analysis of sex and age in hepatocellular carcinoma: a SEER study

**DOI:** 10.1097/MEG.0000000000002745

**Published:** 2024-03-04

**Authors:** Jun Chen, Xiao Wang, Wenyi Ye

**Affiliations:** a Department of Geriatrics, The First Affiliated Hospital of Zhejiang Chinese Medical University (Zhejiang Provincial Hospital of Chinese Medicine); b Department of Traditional Chinese Internal Medicine, The First Affiliated Hospital of Zhejiang Chinese Medical University (Zhejiang Provincial Hospital of Chinese Medicine), Hangzhou, China

**Keywords:** end results database, epidemiology, hepatocellular carcinoma, overall survival, prognosis, sex, surveillance

## Abstract

**Objectives:**

This study aimed to explore the impact of sex on clinical features and survival among hepatocellular carcinoma (HCC) patients.

**Methods:**

HCC case data from the Surveillance, Epidemiology, and End Results (SEER) database for the period 2010 to 2015 were selected for analysis. Kaplan–Meier curves displayed overall survival. Univariate cox regression examined the prognostic characteristics of individual features, and multivariate Cox regression assessed hazard ratios.

**Results:**

This study comprised 3486 HCC patients, with 2682 males and 804 females. Across all age groups, there was a higher prevalence of males compared to females. Survival curves among female patients showed no significant differences across various age groups. However, among male patients, those under 60 demonstrated notably higher survival rates compared to those aged 60 and above. Regarding various ethnicities, TNM staging systems, tumor sizes, the presence of lung/bone/brain metastases, location in Purchased/Referred Care Delivery Areas, SEER historic stages, tumor grades, and individuals receiving chemotherapy, the proportion of male patients consistently exceeded that of female patients. Within the female patient group, individuals receiving chemotherapy exhibited significantly higher survival rates compared to those who did not. However, the administration of chemotherapy showed no significant impact on the survival rate of male patients. Multivariate Cox regression analysis revealed age, gender, and the administration of chemotherapy key factors influencing the overall survival prognosis.

**Conclusion:**

Age, gender, and the administration of chemotherapy are influential factors in the prognosis of both male and female HCC patients.

## Introduction

Liver cancer is the leading cause of global cancer-related deaths and ranks fifth in the USA. Over 90% of liver cancer cases are attributed to hepatocellular carcinoma (HCC) [1]. Major causes of HCC include infection with hepatitis B virus (HBV) and hepatitis C virus, heavy alcohol consumption, intake of aflatoxin B1, smoking, and nonalcoholic fatty liver disease (NAFLD) resulting from obesity and insulin resistance [2]. Due to the implementation of HBV vaccines and direct-acting antivirals, NAFLD is projected to become the predominant cause of HCC in numerous countries by 2030 [3]. NAFLD can progress from simple steatosis to nonalcoholic steatohepatitis, liver fibrosis, and cirrhosis, and eventually, HCC [4]. Currently, the prognosis of HCC is unfavorable, making the exploration of factors influencing HCC prognosis a prominent research topic.

The Surveillance, Epidemiology, and End Results (SEER) database is a nationally representative, population-based cancer reporting system that includes all cancer cases within a specific geographic area of the USA. Analyzing longitudinal trends in cancer diagnosis, treatment, and survival through SEER contributes to a more intuitive understanding of cancer diagnosis and treatment [5]. Numerous studies have demonstrated the utility of the SEER database for survival and prognosis analysis of HCC. For example, Lin *et al*. investigated the impact of liver fibrosis on microvascular invasion and the prognosis of HCC with isolated nodules using the SEER database [6]. Zhu *et al*. identified clinicopathological factors influencing survival after hepatectomy in patients with early HCC and hepatitis B virus-associated cirrhosis using the SEER database [7]. The evidence above suggests that the SEER database is a commonly used and reliable tool for discussing HCC prognosis.

Numerous studies have demonstrated that sex is one of the key factors influencing cancer survival and prognosis, including colon [8], stomach [9], and esophageal cancers [10]. Research has shown that the majority of HCC patients are male, with a male-to-female ratio ranging between 1.5 and 4.0 [11,12], indicating that sex may also be a crucial factor influencing the prognosis of HCC patients. Additionally, the clinical features of tumors differ between men and women [11]. Therefore, we downloaded clinical data of HCC patients from the SEER database spanning from 2010 to 2015 and explored the impact of clinical features of HCC patients on survival and prognosis in both men and women.

## Methods

### Data download

A retrospective cohort analysis of patients with HCC was performed using the SEER (https://seer.cancer.gov/) database, covering 3486 cases from 2010 to 2015. Patients were excluded if their survival time and survival status were not available. Demographic and clinicopathologic information, including age at diagnosis, race, Purchased/Referred Care Delivery Areas, SEER historic stage, tumor size [determined by computed tomography (CT)], T stage (T1, T2, T3, T4), N stage (N0, N1, N2), M stage (M0, M1), presence of brain, bone, and lung metastases, whether the patient received chemotherapy or not, and survival follow-up (survival time and survival status) were downloaded using SEER*Stat software.

The cancer diagnosis, staging, and metastatic information in the SEER database mainly originate from medical records, pathology reports, cancer registries, and records of radiotherapy. HCC diagnosis in our study cohort primarily emanates from pathology reports obtained from medical institutions. Pathologists thoroughly examine tissue samples to ascertain the presence of HCC, identify its specific type, and document other pertinent pathological features. Subsequently, this information is recorded in the patients’ medical records and reported to cancer registries. Information on HCC staging is mainly derived from physicians’ diagnoses, surgical records, and relevant imaging studies. The TNM staging system, is used to assess the extent of tumor involvement, lymph node involvement, and metastasis. The identification of distant metastases relies on clinical examinations, imaging studies (such as MRI, CT, and PET scans), and observations made during surgical procedures. The SEER database collects, integrates, and standardizes clinical information from diverse medical records.

### Statistical analysis

We used the age of 60 years as a threshold to define the younger and older age groups. In the survival status data, ‘alive’ was coded as 0, and ‘death’ was coded as 1. For the remaining data without clear classification, we uniformly labeled it as ‘Unknown’. We assessed the survival prognostic characteristics of male and female patients across various levels of clinical characteristics. Chi-square tests were utilized to analyze the relationship between sex classification and various clinical features. Features showing significant differences in the Chi-Square test were subsequently subjected to univariate Cox regression analysis to examine their prognostic characteristics. Overall survival (OS) was analyzed using Kaplan–Meier (KM) [13] and differences between survival curves were tested using the log-rank test. Multivariate Cox proportional hazard regression [14] was used to identify the hazard ratios of multiple clinical characteristics prognostic factors. Statistical analysis was performed using R version 4.3.2.

## Results

### Demographic and clinical characteristics of patients with HCC

From 2010 to 2015, a total of 3486 patients with HCC, including 2682 male patients and 804 female patients, were included in this analysis. The demographic and clinical characteristics of patients (males and females) with HCC are described in Table 1.

**Table 1.** The demographic and clinical characteristics of patients (males and females) with hepatocellular carcinoma

	Female		Male		*P*-value
	Count	Percent (%)	Count	Percent (%)	
Age					0.001164
60 years	215	6.17	882	25.30	
60+ years	589	16.90	1800	51.64	
Race					0.003593
Black	85	2.44	299	8.58	
Other	222	6.37	588	16.87	
White	497	14.26	1795	51.49	
PRCDA					0.4905
Not PRCDA	403	11.56	1305	37.44	
PRCDA	401	11.50	1377	39.50	
Grade					0.1962
Grade I	215	6.17	734	21.06	
Grade II	391	11.22	1381	39.62	
Grade III	188	5.39	534	15.32	
Grade IV	10	0.29	33	0.95	
Stage					0.01229
Distant	76	2.18	342	9.81	
Localized	524	15.03	1614	46.30	
Regional	204	5.85	726	20.83	
T					0.1335
T1	426	12.22	1289	36.98	
T2	144	4.13	566	16.24	
T3	169	4.85	612	17.56	
T4	30	0.86	100	2.87	
TX	35	1.00	115	3.30	
N					0.1522
N0	734	21.06	2386	68.45	
N1	39	1.12	174	4.99	
NX	31	0.89	122	3.50	
M					0.009829
M0	735	21.08	2362	67.76	
M1	69	1.98	320	9.18	
Bone					0.2627
No	784	22.49	2592	74.35	
Yes	20	0.57	90	2.58	
Brain					0.1195
No	804	23.06	2670	76.59	
Yes	0	0.00	12	0.34	
Lung					0.04139
No	780	22.38	2555	73.29	
Yes	24	0.69	127	3.64	
Size					0.6316
100–150 mm	79	2.27	251	7.20	
150–200 mm	31	0.89	87	2.50	
200+ mm	71	2.04	277	7.95	
50-mm	416	11.93	1354	38.84	
50–100 mm	207	5.94	713	20.45	
Chemotherapy					0.01465
No/unknown	539	15.46	1669	47.88	
Yes	265	7.60	1013	29.06	

M, distant metastasis; M0, cancer has not spread to other parts of the body; M1, cancer has spread to other parts of the body; N, regional lymph nodes; N0, there is no cancer in nearby lymph nodes; N1, N2, N3, Refers to the number and location of lymph nodes that contain cancer; NX, cancer in nearby lymph nodes cannot be measured; PRCDA, Purchased/Referred Care Delivery Area; T, primary tumor; T1, T2, T3, T4, Refers to the size and/or extent of the main tumor; TX, main tumor cannot be measured.

Regarding age at diagnosis, 822 (25.30%) male patients were younger than 60 years old, and 1800 (51.64%) were older than 60 years. For female patients, 6.17% were younger than 60 years old, and 16.90% were older than 60 years, significantly lower than their male counterparts. As for patients with different ethnicities, among the Black (males: 8.58%, females: 2.44%), White (males: 51.49%, females: 14.26%), and other populations (males: 16.87%, females: 6.37%), the proportion of male patients was higher than that of female patients. The proportions of male patients in different TNM staging systems and with varying tumor sizes were higher than those of female patients. Additionally, the proportion of male patients (39.50%) in Purchased/Referred Care Delivery Areas was greater than that of female patients (11.50%). As for tumor metastasis, the proportion of bone metastasis, brain metastasis, and lung metastasis in men was 2.58%, 0.34%, and 3.64%, respectively, which were higher than that in women (0.57% for bone metastasis, 0% for brain metastasis, and 0.69% for lung metastasis). Regarding the SEER historic stage, the proportion of men with distant (9.81%), localized (46.30%), and regional (20.83%) spread was higher than that of women (2.18%, 15.03%, and 5.85%, respectively). Regarding tumor grade, the proportion of early-grade (I, II, III, and IV) patients in men was 21.06%, 39.62%, 15.32%, and 0.95%, respectively, higher than the proportion of female patients (I: 6.17%, II: 11.22%, III: 5.39%, and IV: 0.29%). Moreover, the proportion of male patients who received chemotherapy was 29.06%, significantly higher than that of females at 7.60%.

### Survival prognosis analysis

**Fig. 1.**
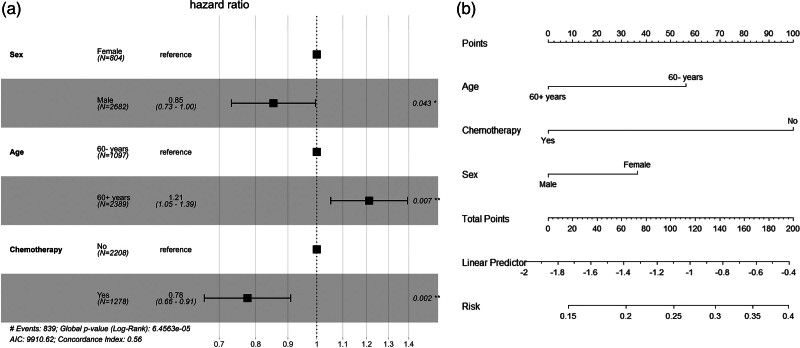
Multivariate Cox proportional hazard regression analysis of overall survival in the retrospective cohort. (a) Multivariable cox regression forest plot. (b) Multivariable cox regression column chart.

To further investigate the influence of clinical features on survival between different genders, we conducted a univariate Cox hazard analysis to identify significant factors, followed by constructing a multivariate Cox regression model. Table 2 highlights that three clinical features, namely age, gender, and the administration of chemotherapy, significantly impact survival (*P* < 0.05). The results from the multivariate Cox regression analysis underscored the statistical significance of age, gender, and chemotherapy administration (*P* < 0.05; Fig. 1a), indicating that these factors were independent and significant predictors when forecasting the survival of HCC patients. Figure 1b illustrates that the administration of chemotherapy had the most pronounced effect on patient survival, while gender exhibited a comparatively smaller impact. Among these factors, females displayed a higher probability of survival compared to males, patients under the age of 60 had a greater likelihood of survival than those older than 60 years, and patients who did not receive chemotherapy exhibited a higher survival rate than those who did.

**Table 2.** Univariate Cox hazard analysis among male and female patients with hepatocellular carcinoma

Characteristic	HR	95% CI	*P*-value
Age			0.004699
60 years	—	—	
60+ years	1.22	1.06, 1.41	
Race			0.7669
Black	—	—	
Other	1.01	0.79, 1.29	
White	0.96	0.76, 1.20	
Stage			0.7217
Distant	—	—	
Localized	0.88	0.51, 1.53	
Regional	0.82	0.46, 1.47	
M			0.2956
M0	—	—	
M1	1.43	0.76, 2.69	
lung			0.3358
No	—	—	
Yes	1.53	0.68, 3.42	
Chemotherapy			0.001508
No	—	—	
Yes	0.78	0.66, 0.91	
Sex			0.03704
Female	—	—	
Male	0.85	0.73, 0.99	

CI, confidence intervals; HR, hazard ratio; M, distant metastasis; M0, cancer has not spread to other parts of the body; M1, cancer has spread to other parts of the body.

### KM survival curves construction

**Fig. 2.**
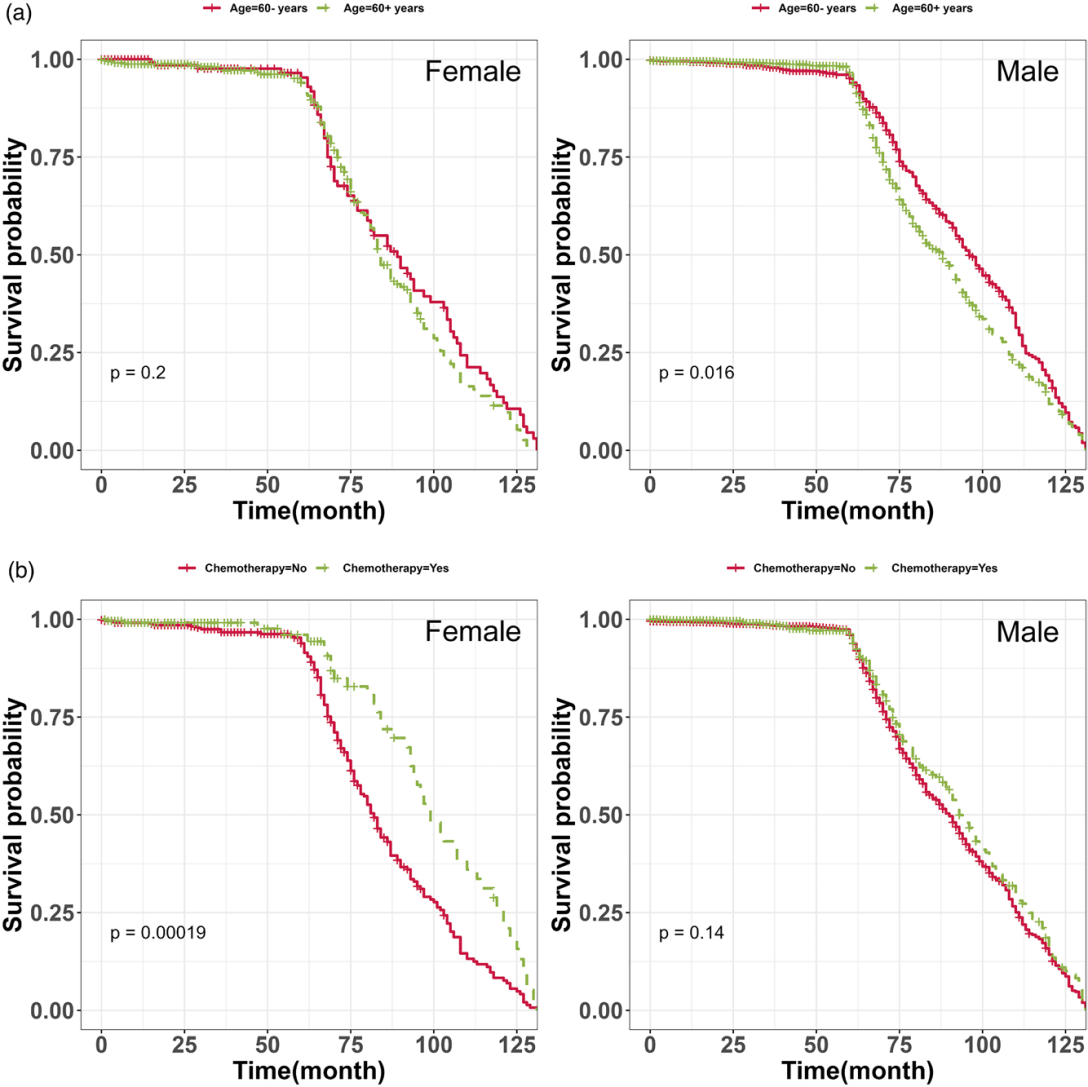
Kaplan–Meier survival curves for overall survival analysis of female (left) and male (right) patients stratified by age at diagnosis (a) and (b) the administration of chemotherapy.

Based on the above analysis, we have identified two features that were both associated with gender and HCC prognosis (chemotherapy and age). To further elucidate the impact of these two features on the survival prognosis of HCC patients, we constructed individual feature KM curves within different genders (Fig. 2a and b). No significant difference was observed in survival curves among female patients across different age groups (Fig. 2a). However, among male patients, the survival rate of those under the age of 60 was significantly higher than that of patients aged 60+ years (*P *= 0.016; Fig. 2a). Figure 2b illustrates that among female patients, the survival rate of those who received chemotherapy was significantly higher than that of those who did not receive chemotherapy (*P* = 0.00019). In contrast, among male patients, no significant difference was observed (Fig. 2b).

## Discussion

Most cancers exhibit sex differences in incidence, mortality, and prognosis [15–18]. Therefore, sex differences are critical issues that cannot be overlooked in oncology research. A previous systematic review noted that women have a survival advantage over men in many cancers. Among the various sex differences observed in cancer manifestations, researchers have noted that some may be associated with occupational and environmental exposures in men [19]. However, in numerous cases, the sex differences observed in many cancers remain unexplained, even when controlling for environmental and genetic factors [20,21]. Therefore, our research focuses on analyzing the effects of clinical indicators on the survival and prognosis of HCC patients of different sexes. Our results suggest that age, gender, and the administration of chemotherapy are factors affecting the prognosis of HCC in different sexes.

We found that the proportion of men with HCC younger than 60 years was higher than the proportion of women (25.30% vs. 6.17%); the proportion of men > 60 years of age with HCC was higher than that of women (51.64% vs. 16.90%) as well. Differences in the age of HCC patients may be related to the body’s hormone levels. The estrogen receptor of hepatocytes has been reported to inhibit the replication and transcription of the hepatitis virus and the release of interleukin-6, a protein associated with chronic hepatitis [22]. Conversely, the androgen receptor plays an opposing role: its activation can enhance the replication of the hepatitis virus and promote the expression of genes related to liver cancer development [23]. Numerous epidemiological studies have linked a lower risk and higher survival rate to increased estrogen exposure in adult females [24,25]. Selecting age 60 as the cutoff point ensures that patients older than 60 years are postmenopausal [8], further highlighting the significance of hormone levels in both sexes. Additionally, various other factors, including epigenetic and genetic factors, sex hormones, and psychosocial aspects, contribute to the observed differences. These factors result in females having a stronger innate and adaptive immune response, thereby reducing the risk of cancer-related mortality [26]. Furthermore, survival analysis revealed that among male patients, those under the age of 60 exhibited a significantly higher survival rate than their counterparts older than 60 years. Moreover, multivariate regression analysis demonstrated that the hazard ratios for age and gender in HCC patients were 1.21 (reference value 1.05–1.39) and 0.85 (reference value 0.73–1.00), respectively, further confirming the pivotal role of age and gender in influencing HCC prognosis.

Chemotherapy is one of the most important treatment modalities for advanced HCC [27]. Our research findings revealed that the proportion of male patients receiving chemotherapy was 29.06%, significantly higher than that of females at 7.60%. This finding aligns with previous results [28]. Furthermore, multivariate Cox regression analysis indicated that the hazard ratio for HCC patients receiving chemotherapy was 0.78 (reference value 0.66–0.91). Kaplan–Meier survival curves demonstrate a significantly higher survival rate for female patients who received chemotherapy compared to those who did not, whereas no significant difference was observed among male patients. In a study by Kaif *et al*. based on the SEER database, HCC patients who received chemotherapy also exhibited a significantly longer median OS compared to those who did not receive chemotherapy [29]. These findings aligned with our research results, and collectively, they supported the notion that the decision to receive chemotherapy was one of the crucial factors influencing the prognosis of HCC.

In this study, we explored the impact of clinical features on HCC survival and prognosis in different sexes, but some shortcomings remain. First, this study was primarily based on observational clinical data and lacks experimental validation. Further experimental research was needed to confirm whether the observed associations have causal relationships. Additionally, due to the limitations of the data, certain important biological or molecular factors were not included in the analysis, which could potentially have an impact on the results.

In conclusion, our study revealed that age, gender, and the administration of chemotherapy were important factors affecting the prognosis of male and female HCC patients. Our research provides a theoretical basis for clinical management and has the potential for clinical applications.

## Acknowledgements

None.

### Conflicts of interest

There are no conflicts of interest.
